# 环状RNA CircHIPK3通过miR-379调控IGF1表达促进非小细胞肺癌细胞系NCI-H1299与NCI-H2170的细胞增殖

**DOI:** 10.3779/j.issn.1009-3419.2017.07.04

**Published:** 2017-07-20

**Authors:** 芳 田, 云 王, 哲 肖, 学军 朱

**Affiliations:** 1 361000 厦门，厦门大学生命科学科学院，细胞应激生物学国家重点实验室 State Key Laboratory of Cellular Stress Biology, School of Life Sciences, Xiamen University, Xiamen 361000, China; 2 210029 南京，南京中医药大学附属江苏省中医院呼吸科 Dpartment of Respiratory Medicine, Affiliated Hospital of Nanjing University of Chinese Medicine, Nanjing 210029, China

**Keywords:** 肺肿瘤, 环状RNA, CircHIPK3, 增殖, Lung neoplasms, Circular RNA, CircHIPK3, Proliferation

## Abstract

**背景与目的:**

已有的研究证明：环状RNA是一类在哺乳动物中普遍存在的具有稳定闭合环状结构的内源性RNA分子。环状RNA circHIPK3（circular RNA HIPK3, circHIPK3）在肝细胞癌（hepatocellular carcinoma, HCC）中表达水平较高，促进肝癌细胞生长。但是其在非小细胞肺癌（non-small cell lung cancer, NSCLC）中的作用及其调控机制尚无文献报道。本研究拟探讨环状RNA circHIPK3对NSCLC细胞系NCI-H1299和NCI-H2170细胞增殖的影响，并进一步研究其调控的分子机制。

**方法:**

Real-time PCR法检测circHIPK3在NSCLC各细胞系中的表达水平。CCK-8实验和克隆形成实验检测过量表达和干扰circHIPK3对细胞增殖的影响。双荧光素酶报告基因实验分别检验miR-379与circHIPK3及miR-379与IGF1 mRNA的结合情况。Western blot和ELISA检测细胞内外的IGF1蛋白表达水平。

**结果:**

环状RNA circHIPK3在6株NSCLC细胞株中均有表达，其中H1299表达最低，H2170表达最高，通过转染过表达的circHIPK3可显著促进NCI-H1299细胞增殖，干扰circHIPK3可显著抑制NCI-H2170细胞增殖。miR-379可与circHIPK3及IGF1 mRNA直接结合。过表达circHIPK3导致IGF1表达量上调，干扰circHIPK3能够下调IGF1表达水平，转入miR-379 mimics恢复了circHIPK3稳转细胞株IGF1表达水平的上调及细胞增殖表型。

**结论:**

环状RNA circHIPK3在NSCLC细胞系NCI-H1299及NCI-H2170中可通过miR-379调控IGF1表达促进细胞增殖，环状RNA circHIPK3可能成为非小细胞肺癌治疗的新靶点。

## Abstract (en)

**Background and objective:**

It has been proven that the circular RNA, possessing a stable covalently closed continuous loop, is a type of RNA molecule which is expressed widespread in mammals. The circular RNA circHIPK3 is abundantly expressed in hepatocellular carcinoma (HCC) and promotes tumourgenesis. However, a role for circHIPK3 has not been systematically examined in non-small cell lung cancer (NSCLC). In this study, we investigated whether circHIPK3 has an effect on cell proliferation in the NSCLC cell lines NCI-H1299 and NCI-H2170 and the underlying molecular mechanisms.

**Methods:**

The expression of circHIPK3 was measured by real-time PCR in NSCLC cell lines. Nuclear mass separation experiment was used to detect the location of circHIPK3 in cells. The gain and loss function experiments were used to examine the proliferation of NCI-H1299 and NCI-H2170 cells by Cell Counting Kit-8 (CCK-8) and the colony formation assays. Then, circHIPK3 was cloned into the downstream of the luciferase reporter gene which activity was detected to verify whether miR-379 could bind with circHIPK3 or IGF1 mRNA. The protein level of IGF1 was detected by Western blot and ELISA in circHIPK3 overexpressed/knock-down NCI-H1299 and NCI-H2170 cells.

**Results:**

CircHIPK3 was generally expressed in six kinds of NSCLC cells lines we detected, and the expression level was highest in H2170 and lowest in H1299. Overexpression of circHIPK3 obviously promoted NCI-H1299 cell proliferation and knock-down of circHIPK3 inhibited NCI-H2170 cell proliferation. In the luciferase assay, miR-379 was observed to sequester circHIPK3 and IGF1 mRNA with potential binding sites. Furthermore, we found that the overexpression of circHIPK3 could increase the expression levels of IGF1 and knock-down reduced it. Moreover, up-regulation of miR-379 rescued the phenotype induced by overexpression of circHIPK3.

**Conclusion:**

CircHIPK3 could promote cell proliferation by a circHIPK3/miR-379 pathway in NCI-H1299 and NCI-H2170 cells and might be a potential tumor biomarker for NSCLC.

肺癌是世界上发病率和死亡率较高的肿瘤之一，其中非小细胞肺癌（non-small cell lung cancer, NSCLC）的发病人数约占肺癌的85%^[1, 2]^。虽然对NSCLC的相关治疗已经取得了一定程度的进展，但是肺癌的5年生存率依然低于15%^[3, 4]^。因此，研究其分子机制及发现新的治疗靶点对肺癌治疗至关重要。

环状RNA（circular RNA, circRNA）是一类广泛存在于哺乳动物体内的非编码RNA，主要参与生物体内基因调控^[5-7]^。CircRNA大部分来源于基因的外显子区域，也有少部分由内含子剪接形成^[8, 9]^。与长链非编码RNA（long noncoding RNA, lncRNA）及microRNA（miRNA）不同之处在于，它们不具备5’端和3’端结构，而是由共价闭合的环状结构形成^[10]^。CircRNA广泛地参与到人类的生理和病理调控的过程中。环状RNA可以通过：①作为miRNA“海绵体”（miRNA sponge）；②与蛋白结合互作；③和翻译成多肽等多种的方式发挥功能，在肿瘤研究中，环状RNA作为miRNA“海绵体”调控下游靶基因的机制已经被广泛报道^[11-13]^。目前发现多个环状RNA自身包含至少一个miRNA结合位点，因此，其可以作为RNA的“海绵体”吸附miRNA，从而通过竞争性内源RNAs（competing endogenous RNA, ceRNAs）的机制调控被miRNA抑制的下游靶基因的表达^[11]^。竞争性内源RNAs理论在2010年由Pandolfi等提出，研究发现在细胞中的长链非编码RNA、假基因RNA和环状RNA与编码蛋白的mRNA包含共同的可以被miRNA结合的位点，通过诱饵或“海绵”吸附的机制竞争性的结合miRNA，导致这些不同类型的RNA之间形成相互调控的网络，在细胞的各个生理过程中发挥重要的功能^[14]^。在现有研究^[15]^报道中，环状RNA ciRS-7和circFOXO3等都可以通过ceRNA的机制调控肿瘤细胞的发生与发展。环状RNA HIPK3（circular RNA HIPK3, circHIPK3）在肝、脑、肺中高表达，其主要起源于基因*HIPK*3的第二个外显子。HIPK3起源的环状RNA有3种剪接体，分别为circHIPK3、circHIPK3.1和circHIPK3.2。但是只有circHIPK3丰度较高，并且在细胞中具有显著的功能。研究发现circHIPK3可以结合多个miRNA，包括miR-124和miR-379等。在肝癌中，circHIPK3可以作为“海绵体”吸附miR-124，通过ceRNA机制上调miR-124下游靶基因*IL6R*和*DXL2*，从而促进肝癌细胞的增殖^[16]^。但NSCLC中环状RNA HIPK3的表达及调控机制尚未明晰。

此外，miR-379是抑制肿瘤细胞增殖的一类miRNA。在NSCLC中，miR-379可以增加肿瘤细胞对顺铂的敏感性，增强顺铂的杀伤能力^[17]^。研究表明在血管平滑肌细胞中，miR-379可以通过调控类胰岛素样生长因子（insulin like growth factor, IGF1）从而达到抑制了细胞增殖、侵袭与转移的作用。IGF1在前列腺癌、乳腺癌、肺癌等多种肿瘤细胞中高表达，促进肿瘤细胞的增殖^[18]^。本研究中，主要报道了circHIPK3在NSCLC细胞系NCI-H1299和NCI-H2170中的功能，并初步提出circHIPK3可以通过miR-379/IGF1促进NSCLC细胞系NCI-H1299和NCI-H2170细胞增殖的机制，希望能为NSCLC治疗提供了一个新的治疗靶点。

## 1 材料与方法

### 1.1 材料

NCI-H1299和NCI-H2170细胞株（购自ATCC细胞库）；BCA蛋白含量检测试剂盒（购自北京全式金有限责任公司）；RPMI-1640、高糖DMEM、胰酶（购自美国GIBCO公司）；荧光定量Real-Time PCR试剂盒、点突变试剂盒（购自南京诺唯赞公司）；RNA提取试剂Trizol、逆转录试剂盒、Lipo2000、DMSO、DEPC水（购自美国Invitrogen公司）；引物（购于上海生物工程公司），IGF1多克隆兔抗（购于美国Abcam公司）；山羊抗兔β-actin单克隆抗体、HRP标记的羊抗兔IgG、双萤光素酶报告基因检测试剂盒（购自南京碧云天公司）；PVDF膜（购自美国Bio-Rad公司）；ECL化学发光试剂盒（购自美国PS公司)；核质分离试剂盒（购于美国Ambion公司）；miR-379 mimics、miR-379 mimics control、miR-379 inhibitor和miR-379 inhibitor control（购自广州锐博公司）；CCK-8试剂（购自日本同仁化学研究所）；Modulus^TM^单管型多功能检测仪（购自美国Promega公司）；IGF1 ELISA检测试剂盒（购于美国Life公司）。

### 1.2 方法

#### 1.2.1 细胞培养

NCI-H1299和NCI-H2170细胞系的培养液成份为10%胎牛血清和RPMI-1640培养基，培养液含青霉素/链霉素100 U/mL。细胞培养于37 ℃、含5%CO_2_培养箱中，0.25%的胰酶常规消化，选择生长较好的对数期进行实验。

#### 1.2.2 质粒构建及细胞转染

环状RNA过表达载体pLCDH-circRNA购广州自吉赛生物公司，序列circHIPK3（hsa_circ_0000284）来自数据库cirBase。用Fast pfu扩增circHIPK3序列，克隆构建过表达质粒pLCDH-circHIPK3。circHIPK3序列片段插入荧光素酶报告基因下游，构建包含circHIPK3的报告基因质粒pGL3-circHIPK3。采用点突变试剂盒突变circHIPK3与miR-379结合位点。pLCDH-circHIPK3慢病毒转染法感染细胞，48 h后以1 μg/mL浓度的嘌呤霉素筛选细胞株，鉴定稳转细胞系。实验组均设3个复孔。干扰siRNA购自锐博生物公司。除过表达外，其余质粒和siRNA通过脂质体法转入NCI-H1299或NCI-H2170细胞中，通过GFP荧光检测转染效率，所有质粒都通过测序鉴定。

#### 1.2.3 核质分离实验及RNase R消化线性RNA实验

将NCI-H1299和NCI-H2170细胞分别以2×10^5^个/孔铺入6孔板各3个孔，当细胞处于80%融合度时用0.25%的胰酶消化细胞，2, 000 *g*离心2 min，根据核质分离试剂盒步骤，分别回收胞质和胞核RNA，nano 2000测定含量，储存待用。将RNA分为RNase消化组和非消化组两组，准备10×Reaction Buffer配制10 μL总反应体系，用于消化线性RNA，每1 μg RNA用1 U（1个单位）的RNase消化，37 ℃ 10 min。随后用苯酚/氯仿、乙醇沉淀法提取消化产物，逆转录为cDNA，通过实时荧光定量PCR法检测circHIPK3在核质中的表达，实验共重复三次。

#### 1.2.4 实时荧光定量PCR检测RNA表达水平

抽提样品RNA，经浓度和纯度测定后，逆转录合成cDNA样品，以β-actin为内参。将SYBR Green预混液、模板、上/下游引物、ddH_2_O配制成PCR反应溶液，置于Real-time PCR仪上进行PCR扩增反应。反应条件为：95 ℃ 2 min预变性，然后按95 ℃ 1 min，60 ℃ 1 min，72 ℃ 1 min，共40个循环，最后72 ℃ 7 min延伸。结果通过2^-△△CT^法分析基因相对表达量。PCR引物序列见表 1。

**1:** PCR引物序列 The PCR primers sequence

Item	Sequence
circHIPK3-F	cggaattcTGAAATATGCTATCTTACagGTATGGCCTCACAAGTCTTG
circHIPK3-R	cgggatccTCAAGAAAAAATATATTCacCTGTAGTACCGAGATTGTAG
circHIPK3 point mutation primer F	GTCTTGGTGATGCCACCATATGTTTATCAAACTCAGTCAAG
circHIPK3 point mutation primer R	ATATGGTGGCATCACCAAGACTTGTGAGGCCATAC
si-circHIP3-1	ACUACAGGUAUGGCCUCACAA
si-circHIP3-2	CUACAGGUAUGGCCUCACA
si-circHIP3-3	UACAGGUAUGGCCUCACAAGU

#### 1.2.5 CCK-8检测细胞增殖实验

NCI-H1299和NCI-H2170细胞增殖检测实验主要通过CCK-8试剂盒完成。实验分为对照组和处理（过表达和敲低）组，采用96孔板中，每个孔铺大约1×10^3^个细胞，培养5 d。加入CCK-8后，37 ℃孵育2 h，检测OD_450_的值。

#### 1.2.6 平板克隆形成实验

实验分为对照组和NCI-H2170 circHIPK3沉默组，对照组和NCI-H1299 circHIPK3过表达组，每组按每孔接种约5×10^2^个细胞于6孔板中，37 ℃培养两周后，用结晶紫染色计数，每个实验重复3个孔。

#### 1.2.7 双荧光素报告基因实验

实验分为6组：pGL-3空质粒组、pGL-3-circHIPK3组、circHIPK3突变组、circHIPK3 siRNA组、miR-379 inhibitor组及miR-379 inhibitor control组。每组中都转染海肾萤光素酶内参质粒和miR-379 mimics。转染24 h后吸尽细胞培养液，根据试剂盒要求加入适量裂解液充分裂解细胞。10, 000 *g*离心5 min后，取裂解液上清100 μL用于测定。以海肾萤光素酶为内参，用萤火虫萤光素酶测定得到的RLU值除以海肾萤光素酶测定得到的RLU值。根据得到的比值来比较不同样品目的报告基因的激活程度。

#### 1.2.8 Western blot检测蛋白表达水平

在收集的蛋白样品中加入适量浓缩的蛋白上样缓冲液，100 ℃沸水浴加热3 min-5 min，以充分变性蛋白。经过跑胶、转膜、封闭、一抗、二抗及显影液孵育后，于Bio-Rad公司化学发光成像仪显影成像。

#### 1.2.9 IGF1 ELISA检测

将NCI-H1299细胞分为转染miR-379 mimics control和过表达miR-379 mimics、miR-379 inhibitor control及miR-379 inhibitor两组，以1 mL培养基重悬2×10^5^个细胞为一孔，加入24孔板，每组做3个复孔。细胞培养72 h后，采用无菌管收集上清于3, 000 *g*离心20 min。根据试剂盒说明，用纯化的IGF1抗体4 ℃包被96孔酶标板，并放置过夜。第2天封闭过后，往包被单抗的微孔中依次加入100 μL不同倍比稀释度的细胞上清，并加入用细胞培养基梯度稀释的对照样品，37 ℃孵育2 h。PBST洗涤5次，加入100 μL稀释后的HRP标记的二抗，37 ℃孵育1 h。PBST再次洗涤，显色剂显色20 min后终止反应，用酶标仪在450 nm波长下测定吸光度（OD值），通过标准曲线计算样品中人IGF1浓度，结果以差异倍数显示。

### 1.3 统计学方法

数据通过Graphad Prism软件作图和统计，以均数±标准差（Mean±SD）表示。实验均采用两独立样本*t*检验，以*P*＜0.05为差异有统计学意义。

## 2 结果

### 2.1 鉴定circHIPK3在NSCLC细胞系中的表达

首先分别以细胞NCI-H1299的基因组DNA（gDNA）和cDNA作为模板，通过PCR技术扩增circHIPK3包含成环位点（back spliced junction）的片段，结果表明模板为cDNA组的PCR产物出现约100 bp的目的条带，并且基因组DNA不能非特性扩增circHIPK3（图 1A）。Sanger测序结果验证了成环位点序列（图 1B）。利用RNase R消化细胞NCI-H1299的RNA，qRT-PCR结果显示RNase R处理前后，circHIPK3表达水平无明显变化，但是线性mHIPK3表达水平却显著降低（图 1C，*P*＜0.01）。进一步通过细胞核质分离实验证明circHIPK3主要存在于NCI-H1299和NCI-H2170的细胞质（图 1D）。

**1:**
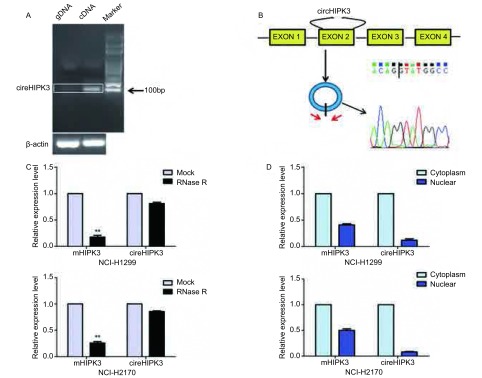
CircHIPK3在NSCLC细胞系中的鉴定。A：分别以基因组DNA和cDNA为模板，PCR扩增circHIPK3成环位点附近100 bp序列，白色矩形框表示目的条带区域；B：circHIPK3成环示意图及测序结果，红色箭头表示divergent primers，竖短黑线示意成环位点；C：RNase R消化后的mHIPK3和circHIPK3的丰度检测；D：circHIPK3在细胞核中与细胞质中的含量比较。^**^：与对照组相比，*P*＜0.01。 Identification of circHIPK3 in NSCLC cell lines. A: CircHIPK3 sequence about 100 bp around back splied junction was analyzed by PCR, and the white rectangle indicated DNA bands; B: Schematic diagram of circular point. Red arrows represented divergent primers, and vertical short black line indicated circular point; C: The levels of circHIPK3 and mHIPK3 were analyzed after RNase R digestion; D: Expression of circHIPK3 in both nuclear and cytoplasmic fractions were measured by qRT-PCR. ^**^: compared with the control, *P* < 0.01.

### 2.2 过量表达circHIPK3促进NCI-H1299细胞的增殖

我们首先在NSCLC细胞系H1299、H827、H1975、H2170、H520、H1650中均检测到circHIPK3的表达，发现NCI-H2170表达量最高，NCI-H1299表达量最低（图 2A）。在NCI-H1299细胞系中稳定转染circHIPK3，circHIPK3表达水平显著上调，mHIPK3无明显变化（图 2B-图 2C）。CCK-8实验结果显示实验组circHIPK3稳定表达株的OD_450_明显高于对照组（图 2D，*P*＜0.01），且其在平板克隆形成实验中的克隆数目多于对照组（图 2E-图 2F，*P*＜0.01）。

**2:**
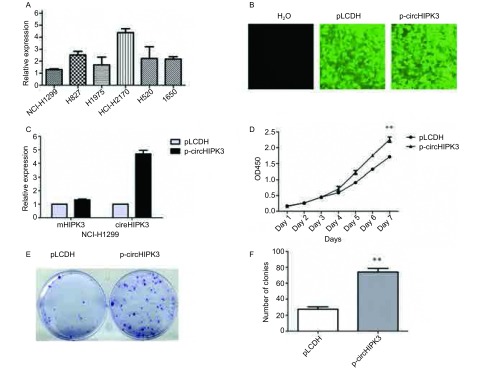
过量表达circHIPK3促进NCI-H1299细胞增殖。A：circHIPK3在6株NSCLC细胞系中的表达量；B：稳定转染对照空质粒pLDCH和circHIPK后，NCI-H1299细胞表达载体携带的绿色荧光蛋白标记；C：过表达circHIPK后，细胞中circHIPK3及mHIPK3表达水平检测；D：CCK-8试验检测细胞增殖；E：平板克隆形成实验检测细胞增殖；F：克隆形成数目统计；^**^：与对照组相比，*P*＜0.01。 Overexpression of circHIPK3 promoted NCI-H1299 cells proliferation. A: The expression level of circHIPK3 in 6 kinds of NSCLC cell lines; B: The green fluorescent protein (GFP) indicated the successful and stable establishment of pLDCH/p-circHIPK3 NCI-H1299 cell line; C: Detection of circHIPK3 and mHIPK3 expression levels in NCI-H1299; D: The cell growth rate was measured by CCK-8 assay; E: Cell proliferation was assessed by colony formation assay; F: Statistics analysis of colony formation assay; ^**^: compared with the control, *P* < 0.01.

### 2.3 沉默circHIPK3抑制NCI-H2170细胞系的增殖

我们订购了三条siRNA干扰circHIPK3的表达，其中si-circHIPK3-2有明显的干扰抑制效果（图 3A）。干扰circHIPK3实验组的OD_450_显著低于对照组（图 3B，*P*＜0.01）。克隆形成数目也明显低于对照组（图 3C-图 3D，*P*＜0.01）。

**3:**
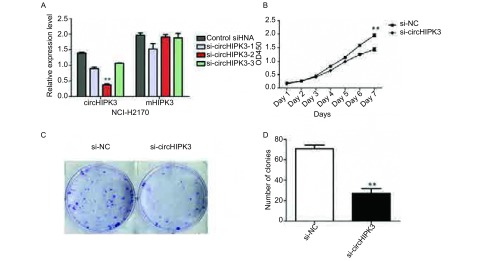
沉默circHIPK3抑制NCI-H2170细胞系的增殖。A：cirHIPK3 siRNA敲降效果检测；B：CCK-8实验检测circHIPK3沉默后对细胞增殖的影响；C：平板克隆形成实验检测circHIPK3沉默后对细胞增殖的影响；D：克隆形成数目统计；^**^：与对照组相比，*P*＜0.01。 Silencing circHIPK3 inhibited NCI-H2170 cell proliferation. A: The knock-down efficiency of cirHIPK3 siRNAs; B: Cell proliferation was evaluated by CCK-8 assay; C: Cell proliferation was detected by colony formation assay; D: Statistics of colony formation assay; ^**^:compared with the control, *P* < 0.01.

### 2.4 circHIPK3作为“海绵体”吸附miR-379

我们将circHIPK3的序列插入荧光素酶报告基因载体pGL3 promoter-luc单位的下游，与miR-379 mimics及内参质粒共转入293T和NCI-H1299细胞中后，相对于载体对照组（EV），实验组circHIPK3的荧光强度显著降低。突变circHIPK3与miR-379的结合位点，将突变质粒Luc-circHIPK3 mutant（mut-circ）与miR-379 mimics共转入293T和NCI-H1299中，荧光信号没有发生明显改变。此外，将si-circHIPK3（si-circ）、miR-inhibitor（miR-in）、Inhibitor control（IC）与miR-379 mimics共转入293T和NCI-H1299中，荧光强度也没有显著降低（图 4A-图 4B）。以上数据表明circHIPK3可以与miR-379结合，作为“海绵体”吸附miR-379。此外，我们同样验证了miR-379与IGF1 3’UTR的结合，双荧光素酶报告基因结果显示，miR-379可以与IGF1 3’UTR直接结合（图 4C-图 4D）。

**4:**
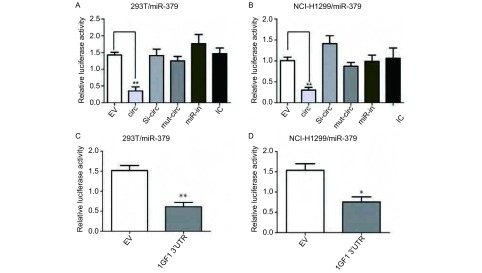
CircHIPK3作为“海绵体”吸附miR-379。A：荧光素酶报告基因实验检测miR-379分别和载体对照组EV、circHIPK3过表达组（circ）、circHIPK3敲低组（si-circ）、突变质粒Luc-circHIPK3组（mut-circ）、miR-inhibitor组（miR-in）及Inhibitor control组（IC）共转入293T细胞中的荧光素酶的相对荧光强度；B：荧光素酶报告基因实验检测miR-379分别和EV、circ、si-circ、mut-circ、miR-in及IC共转入NCI-H1299细胞中的荧光素酶的相对荧光强度；C：转入miR-379 mimics和IGF1 3’UTR后，293T细胞中荧光素酶的相对荧光强度检测；D：转入miR-379 mimics和IGF1 3’UTR后，NCI-H1299细胞中的荧光素酶相对荧光强度。^*^：与对照组相比，*P*＜0.05。^**^：与对照组相比，*P*＜0.01。 CircHIPK3 could sequester miR-379. A: Luciferase relative activity of miR-379 in EV, circ, si-circ, mut-circ, miR-in and IC treated 293T cells; B: Luciferase relative activity of miR-379 in EV, circ, si-circ, mut-circ, miR-in and IC treated NCI-H1299 cells; C: Luciferase relative activity of miR-379 with IGF1 3'UTR in 293T cells; D: Luciferase relative activity of miR-379 with IGF1 3'UTR in NCI-H1299 cells. ^*^: compared with the control, *P* < 0.05. ^**^: compared with the control, *P* < 0.01.

### 2.5 circHIPK3通过circHIPK3/miR-379促进细胞增殖

在NCI-H1299中转入miR-379 mimics后，IGF1表达水平降低，转入miR-379 inhibitor发现IGF1表达水平上调（图 5A-图 5B，图 5E，*P*＜0.05）。在NCI-H2170中干扰抑制circHIPK3，发现IGF1表达量明显下调（图 5C，图 5E，*P*＜0.05）。在过表达circHIPK3的NCI-H1299的细胞系中转入miR-379 mimics后，能部分下调IGF1的表达水平，同时能部分回复对细胞增殖的影响（图 5D，图 5F，*P*＜0.05）。因此，在NCI-H1299和NCI-H2170中，circHIPK3通过miR-379调控IGF1表达促进细胞增殖。

**5:**
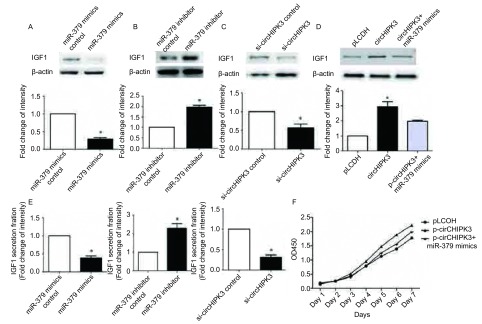
CircHIPK3通过circHIPK3/ miR-379通路促进细胞增殖。A：转入miR-379 mimics后，Western blot检测NCI-H1299细胞内IGF1的表达水平；B：转入miR-379 inhibitor后，Western blot检测NCI-H1299细胞内IGF1的表达水平；C：敲低circHIPK3后，Western blot检测NCI-H2170细胞内IGF1的表达水平；D：分别转染：对照pLCDH、p-circHIPK3、p-circHIPK3+miR-379 mimics共同处理组质粒后，Western blot检测NCI-H1299细胞内IGF1的表达水平；E：细胞培养72 h后，ELISA法检测细胞上清IGF1的量；F：CCK-8实验检测空载质粒pLCDH、p-circHIPK3、p-circHIPK3+miR-379 mimics共同处理组三个组别对细胞增殖的影响。^*^：与对照组相比，*P*＜0.05。 CircHIPK3 promoted NSCLC cell proliferation through circHIPK3/miR-379 pathway. A: The effects of miR-379 mimics on the expression levels of IGF1 in NCI-H1299; B: The effects of miR-379 inhibitor on the expression levels of IGF1 in NCI-H1299; C: The effects of si-circHIPK3 on the expression levels of IGF1 in NCI-H2170; D: The effects of the pLCDH, p-circHIPK3, the group of circHIPK3 combined with miR-379 mimics on the expression levels of IGF1 in H1299; E: Detection of the expression levels of IGF1 by ELISA after culturing for 72 h; F: The cell growth effects of circHIPK3 and the group of circHIPK3 combined with miR-379 mimics on NCI-H1299. ^*^: compared with the control, *P* < 0.05.

## 3 讨论

越来越多的研究显示环状RNA在疾病的发生与发展中起着重要的作用。但是，目前只有CDR1as和circFOXO3研究的较为透彻。circHIPK3是另一个崭露头角的环状RNA，受到人们越来越多的重视。在正常细胞内，其表达丰度与其线性RNA相当，甚至更高。在肿瘤研究中发现，circHIPK3可以促进肝癌的增殖^[16]^。在本文中，我们主要报道了针对circHIPK3在NCI-H1299和NCI-H2170中的功能与初步的机制研究结果。过量表达的circHIPK3同样可以促进NSCLC细胞的增殖，此外，我们的研究更进一步发现，在NSCLC细胞系中，circHIPK3的促进增殖能力可以通过circHIPK3/miR-379发挥，目前研究发现circHIPK3可以与多种miRNA结合，起到miRNA“海绵体”的作用。在肝癌中，circHIPK3可以吸附miR-124，从而调节miR-124的靶基因IL6R和DLX2，促进细胞增殖^[16]^。但是在NSCLC中，circHIPK3同样可以通过吸附miR-379。文献^[18]^报道发现，miRNA-379能够抑制IGF1的表达水平，我们猜测在NSCLC细胞系circHIPK3可能通过吸附miRNA-379促进IGF1的表达，从而影响细胞增殖。Western blot和ELISA证实，过量表达miRNA-379，IGF1的蛋白水平下调。干扰抑制miRNA-379可以上调IGF1的蛋白表达水平。过量表达circHIPK3可以促进IGF1的表达，但是干扰抑制circHIPK3却可以抑制IGF1的蛋白水平。双荧光光素酶报告基因实验表明，miR-379分别可以和circHIPK3及IGF1 mRNA直接结合。在稳转circHIPK3的NCI-H1299细胞系转入miR-379可以挽救circHIPK3过量表达的表型。至此，我们提出circHIPK3在NSCLC细胞系H1299和H2170中促进增殖的机制是通过circHIPK3/miRNA-379实现的。

在细胞系中，我们发现circHIPK3在NCI-H2170高表达，在NCI-H1299低表达。所以，我们选择在NCI-H1299中稳定过表达circHIPK3，在NCI-H2170中干扰抑制circHIPK3。值得注意的是，环状RNA过表达的关键步骤在于成环，我们选用商业化的质粒去过表达circHIPK3，利用Real-time PCR的方法检验circHIPK3的表达量，希望能确定过表达的真实性。因为目前相关体外成环技术并不成熟，成环过程中会形成小部分错误成环的RNA，虽然本研究并没有检验错误成环的影响，但是通过干扰circHIPK3发现细胞的表型与过表达相反，证实细胞增殖的变化确实由circHIPK3的表达量变化引起。为了进一步研究circHIPK3调控NSCLC增殖的分子机制，本研究利用双荧光酶活性实验证明了circHIPK3和miR-379是直接结合，通过文献报道发现，miR-379可以直接结合靶基因*IGF1*的mRNA，抑制IGF1表达水平，从而抑制细胞增殖^[18]^。我们过表达miR-379后，IGF1的表达水平明显下调，说明miR-379在NSCLC细胞中同样可以结合IGF1的mRNA，抑制IGF1表达水平。过表达circHIPK3发现IGF1表达量上调，反之亦然，这就更加确定circHIPK3是可以调控IGF1的上游RNA。虽然文献报道，IGF1是一种重要的细胞生长和分化的调控因子，但是关于IGF1与细胞增殖的关系，本文主要参考相关文献报道，实验上并未做深入探讨，这也是本文的不足之处^[19-21]^。此外，本研究并未进一步证实circHIPK3的调控关系是否能在更多种的细胞系中重复，这一点也是今后研究的重要内容。

综上所述，本研究表明circHIPK3能够促进NSCLC细胞的增殖，circHIPK3通过miR-379调控IGF1表达是促进NSCLC细胞的增殖的机制。在后续的研究中，我们将更加深入地探讨机制，为NSCLC的治疗提供新的思路。
